# 

**Published:** 1905-09-30

**Authors:** 


### NOTICE TO CORRESPONDENTS.

All MSS., Letters, Books for Review, and other matters intended for the Editor should be addressed The Editor, "The Hospital," The Hospital Buildings, 28 & 29 Southampton Street, Strand, W.C.

The Editor cannot undertake to return rejected MS., even when accompanied by stamped directed envelope.

**Notice to Advertisers and Subscribers.**

All Advertisements, Orders for Copies of The Hospital, and Business Communications should be addressed to **THE MANAGER (Not to the Editor),** The Hospital, 28 & 29 Southampton Street, Strand, London, W.C.

The Cost of Subscription, post free, is at follows:—Per week, 3½d.; per quarter, 3s. 3d.; per half-year, 6s. 6d.; per annum, 13s. Foreign and Colonial:—Per annum, 19s. 6d., payable, in all cases, in advance.

**NOTICE.—Vol. XXXVII. of "The Hospital" October 1904 to March 1905), in handsome cloth binding, will shortly be on sale at the office of this Journal, price 10s. 6d. Cases for binding the Numbers contained in this Volume 2s. Index to Vol. XXXVII., 3½d., post free.**

**The Monthly Part for September is now ready. Price 1s., by post, 1s. 3d.**

### BOOKS RECEIVED.

CASSELL AND CO.

"Clinical Methods." New Edition. By Robert Hutchison, M.D., F.R.C.P., and Harry Rainy, M.A., F.R S.E.

"Hygiene and Public Health." New Edition. By Arthur Whitelegge, C.B., M.D.Lond., F.R.C.P., D.P.H., and George Newman, M.D., D.P.H., F.R.S.E.

"A Manual of Chemistry." New Edition. By Arthur P. Luff, M.D.Lond., F.R.C.P., F.I.C., and Frederick James M. Page, B.Sc.Lond., F.I.C.

T. NELSON AND SONS.

"Adam Bede."

ADLARD AND SON.

"St. Bartholomew's Hospital Students' Union Year Book. 1905."

LONGMANS, GREEN, AND CO.

"The Model Kitchen." By Lucy H. Yates

### MEDICAL APPOINTMENTS.

**C. S. Brebner**, M.D., Ch.B.Edin., D.P.H., Medical Officer of Health to the Widnes Town Council. **C. C. Brodrick**, L.R.C.P., L.R.C.S.Edin., reappointed Medical Officer of Health for the Tavistock Rural and Urban Districts, Devon. **John Walter Browne**, M.B., B.S., R.U.Irel., Public Vaccinator at Rawene and Kohukohu, New Zealand. **Edward Seaton Cockell**, M.R.C.S.,

L.S.A., Medical Officer and Public Vaccinator for the Greatham District of Hartlepool. **Robert Warren Crooke,** L.R.C.P.Edin., L.R.C.S.Edin., L.F.P.S.Glasg., Government Medical Officer and Vaccinator at Gunnedah, New South Wales. **Constance E. D'Arcy,** M.B., Ch.M.Syd., Resident Medical Officer, Royal Hospital for Women, Sydney, New South Wales. **B. G. Derry,** M.R.C.S., L.R.C.P.Lond., reappointed Medical Officer of Health for the Borough of Bodmin (Cornwall). **Harold William Litton Harding,** M.R.C.S., L.R.C.P.Lond., Public Vaccinator for the Districts of Upper Hutt, Lower Hutt, and Petone, New Zealand. **E. Henry,** F.R.C.S.Eng., L.R.C.P.Lond., Certifying Surgeon under the Factory and Workhouse Act for the Long Sutton District of the county of Lincoln. **A. J. Laird,** M.D.Glasg., reappointed Medical Officer of Health to the Borough of Crewe. **Lawrie McGavin,** F.R.C.S.Eng., Consulting Surgeon to the Convent of the Assumption, Kensington Square, W. **S. Macvie,** M.B., M.S. Edin., Certifying Surgeon under the Factory and Workshop Act for the Chirnside District of the county of Berwick. **J. C. McWalter,** D.P.H., F.F.P.S.Glasg., M.D.Brux, Examiner in Pharmacy to the Apothecaries' Hall of Ireland. **Wm. Muir,** M.B., C.M.Glasg., Medical Officer to the Post Office in Davidsons Mains District, Midlothian. **P. O'Meehan,** L.D.S.R.S.C.I., Honorary Consulting Dental Surgeon, St. John's Hospital, Limerick. **G. F. Paul,** M.D.Brux, M.R.C.S.Eng., L.R.C.P.Lond., Government Medical Officer and Vaccinator at Cobar, New South Wales. **E. C. Pern,** M.R.C.S., L.R.C.P.Lond., reappointed Medical Officer of Health for the Droxford Urban District Council, Hants. **Leonard Raby,** M.D.Durh., reappointed Medical Officer for the Second District by the Devizes Board of Guardians. **J. Roantree,** M.B., B.S.R.U.I., Certifying Surgeon under the Factory and Workshop Act for the Newbridge District of the county of Kildare. **E. W. Routley,** M.R.C.S., L.R.C.P.Lond., Certifying Surgeon under the Factory and Workshop Act for the Aldershot District of the county of Hants. **Ralph Young,** M.D.Durh., reappointed Medical Officer of Health to the Royton District Council, Durham.

### A PROCLAMATION TO NURSES throughout the Empire

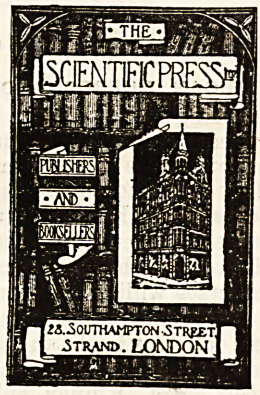

**The Scientific Press desire to inform Nurses everywhere that they are in a position to supply Nursing Literature of all descriptions, Text-Books, Case-Books, Charts, &c. Particulars respecting any publication, promptly sent on receipt of inquiry.**

### SOME NEW BOOKS Ready Shortly.

### SURGICAL INSTRUMENTS AND APPLIANCES USED IN OPERATIONS.

A Classified and Illustrated List, with Explanatory Notes.
By HAROLD BURROWS, F.R.C.S.
Limp cloth, 1/6 net, 1/8 post free.
This work will prove of the utmost service as a work of reference to all Nurses.

### NURSING HINTS TO PROBATIONERS ON PRACTICAL WORK.

By ANNESLEY VOYSEY.
Cloth, Illustrated, 2/- net, 2/4 post free.
This work fills a distinct hiatus in nursing literature. It will prove of great assistance to the Nurse in the early stages of her career.

### MEDICAL ELECTRICITY AND THE LIGHT TREATMENT.

By KATE NEALE,
Sister in Charge Light Department, Guy's Hospital.
Cloth, profusely Illustrated, 2/6 net, 2/9 post free.
A practical book on a subject hitherto untouched from a nursing standpoint.

### THE NURSING OF SICK CHILDREN.

By Dr. BURNET. Cloth, 1/- net, 1/1 post free.
A valuable little treatise on the nursing of children. It should prove useful to mothers as well as to Nurses.

### The Text-Book for the C.M.B. Exam.

### A COMPLETE HANDBOOK OF MIDWIFERY.

By J. K. WATSON, M.D. 6/- net, 6/4 post free.
Illustrated with 143 Diagrams. Cloth gilt.

### A HANDBOOK FOR NURSES.

By J. K. WATSON, M.D.

Cloth, Illustrated, 5/- net, 5/4 post free.

### PRACTICAL GUIDE TO SURGICAL BANDAGING AND DRESSINGS.

By WM. JOHNSON SMITH, F.R.C.S. 2/- post free.
Suitable size for the apron pocket.
Profusely Illustrated.

### SURGICAL WARD-WORK AND NURSING.

By Dr. ALEXANDER MILES, M.D. Edin.
3/6 net, 3/10 post free.
Second Edition. Illustrated. Cloth gilt.

### THE EXAMINATION OF THE URINE.

By J. K. WATSON, M.D. 1/- net, 1/1 post free.
Cloth boards. Suitable size for the apron pocket.

**The Scientific Press, Ltd.**

*Complete Catalogue sent post free on application.*

**28 & 29 Southampton Street, Strand, London, W.C.**

